# Do You Fail to Recognize Me with a Mask on? The Impact of Voice on Mask-Occluded Facial Identity Recognition

**DOI:** 10.3390/bs16010128

**Published:** 2026-01-16

**Authors:** Min Gao, Wenyu Duan, Tianhang Liu, Yulin Gao, Xiaoyu Tang

**Affiliations:** 1 School of Psychology, Liaoning Normal University, Dalian 116029, China; gm4453@lnnu.edu.cn (M.G.);; 2 Huludao First Senior High School, Huludao 125000, China; 3 Department of Philosophy, Jilin University, Changchun 130012, China

**Keywords:** facial identification, mask occlusion, eye-tracking, self-advantage effect, cross-modal facilitation

## Abstract

This research sought to examine differences in the cross-modal facilitation effect of voice on facial identity recognition under mask occlusion for both oneself and others. Employing a facial recognition paradigm, we examined the influence of voice on facial identity recognition under static and dynamic mask occlusion through two eye-tracking experiments. The behavioral results from Experiments 1 and 2 indicate that mask occlusion interfered with recognition for both static and dynamic faces, with greater interference observed for others’ faces than for self-faces. In addition, voice exerts cross-modal enhancement effects on faces, with greater enhancement observed for masked faces than for no mask. Furthermore, voice provides stronger enhancement for others’ dynamic faces than for their self-dynamic faces. Eye-tracking data from both experiments revealed that the difference in dynamic facial recognition between self-faces and others’ faces due to voice emerged in the early stages of dynamic facial recognition and persisted into later stages. However, regardless of whether they were in the early or late stages of static facial recognition, the facilitation effect of voice did not differ between themselves and others. This study revealed that the cross-modal facilitation of visual stimuli by voice is influenced by the self-advantage effect.

## 1. Introduction

Facial identity recognition is one of the most fundamental and complex processes in human cognition and involves the extraction and processing of facial features from visual information. Faces can reveal information about an individual’s identity, gender (Gulati et al., 2022), emotions (Bandyopadhyay et al., 2023), age, and ethnicity (Tsao & Livingstone, 2008), and accurately identifying self-faces is an important step in facilitating children’s development of self-concepts (Cordeiro et al., 2021). Participants respond faster when they recognize self-faces (Devue & Brédart, 2011; Keenan et al., 2000; Sui & Humphreys, 2017; Żochowska & Nowicka, 2024), which is known as the self-advantage effect (Ma & Han, 2010). The self-advantage effect arises not only from differential familiarity with self-faces versus others’ faces but also from the activation of positive attributes in the self-concept; that is, when stimuli associated with the self-concept appear, they automatically activate positive evaluations and emotional connections within the individual’s self-concept. Such activation may enhance social cognitive and emotional responses associated with self-faces, and this enhancement, in turn, facilitates behavioral responses to self-faces, ultimately leading to self-advantages in facial identity recognition (Ma & Han, 2010; Żochowska et al., 2023).

Tatz et al. proposed that information from different sensory modalities can interact with each other; thus, the simultaneous presentation of cross-modality voices can facilitate the recognition of visual targets (Tatz et al., 2020). A previous study suggested that when faces and voices match identities, reaction times are faster and error rates are lower (O’Mahony & Newell, 2012). Even if the voice does not provide information about the location of the face, it can still facilitate visual search for the relevant face (Zweig et al., 2015). Furthermore, individuals can improve their performance in a face gender categorization task by incorporating additional auditory information (Abbatecola et al., 2021; Wen et al., 2023). How does the interaction effect of audiovisual stimuli change when auditory and visual stimuli provide different identity information? Research indicates that we rarely observe the dynamic facial movements associated with our own speech, resulting in weaker facial action memory and the absence of a visual-verbal advantage (Aruffo & Shore, 2012), whereas we view others’ faces alongside voices, such as during a conversation with them. It remains unclear whether the presence of voices differentially influences the recognition of masked facial identity for oneself versus for others. This question has not yet been addressed in prior research. Additionally, it remains unknown whether voice can mitigate the negative effect of mask-occluded faces on the identification of facial identity.

The widespread use of face masks during the COVID-19 pandemic has introduced new challenges to facial identity recognition. The use of masks affects facial identity recognition (Chen & Wang, 2025; Richler et al., 2011; Ritchie et al., 2024), and masks may also introduce additional challenges for social interaction in the general population. Wearing a mask restricts visible facial features to the eye region, which affects how individuals process faces (Carragher & Hancock, 2020; Noyes et al., 2021). Mask occlusion may affect the enhancing effect of voice on facial identity recognition because masks cover the mouth and part of facial expressions, reducing the availability and temporal synchronization of dynamic information. This aligns with foundational research showing that dynamic facial information is critical for the promotional effect of sound on person recognition, especially for familiar person recognition (Schweinberger & Robertson, 2017), suggesting that mask-induced loss of such information may weaken this effect. Under unoccluded mask conditions, individuals process faces holistically, integrating the elements, such as the eyes, nose, and mouth, that make up a face into a whole, a gestalt, for recognition (Maurer et al., 2002; Richler et al., 2011). When unobstructed faces are recognized, holistic processing centred on the nose predominates (Hsiao & Cottrell, 2008; Wang et al., 2013). However, when masked faces are recognized, the mask interferes with facial perception and alters processing mechanisms, making it more reliant on visible features—that is, local processing rather than configurational or holistic information (Freud et al., 2020; Navon, 1977).

The impact of mask occlusion on self- and other-facial identity recognition may differ. Eye-tracking research has revealed that individuals have different fixation positions when recognizing faces of different identities. The representation of one’s own face tends towards characterization and localization rather than holistic depiction (Greenberg & Goshen-Gottstein, 2009). Familiar faces are fixated more on local features, especially the eyes and mouth, whereas unfamiliar faces tend to remain fixated more on the central region of the face (Van Belle, 2010). Additionally, the fixation patterns to self-faces can be task dependent. During free viewing participants tended to focus more on the lower part of their own face than on other familiar and unfamiliar faces did, whereas during identification they relied more on the nose, with little difference across identities (Lee et al., 2022b). The obstruction of masks not only impacts the recognition of other people’s faces but also slows the speed at which individuals recognize their own faces (Żochowska et al., 2022, 2023). Because the processing of self-faces is related to the preferential allocation of attention and emotional responses to self-relevant stimuli, which have greater salience in cognitive evaluation, mask occlusion may interfere with these salient features of self-faces, thereby affecting their recognition (Żochowska et al., 2023).

To investigate the effect of voice on masked facial identity recognition, we conducted two experiments using eye-tracking techniques to dissect behavioral and attentional mechanisms. Experiment 1 focused on an identity judgment task using static images of both self-faces and other faces, accompanied by voice. The aim was to assess the effect of voices on the recognition of static faces covered by masks. Schweinberger suggested that voices enhance the recognition of dynamic faces more effectively than static faces do (Schweinberger & Robertson, 2017). Additionally, dynamic and multisensory input, which is common in the perception of others, interferes with the stable self-representation derived from static mirror views, thus attenuating the self-advantage in facial recognition (Tsakiris, 2008). Consequently, Experiment 2 further investigated the effect of voices on the identification of masked dynamic faces. Eye-tracking technology can further reveal the characteristics of different processing stages in facial identity recognition and the role of voice in facilitating these stages. To investigate the effect of mask obstruction on facial identity recognition processing, we divided the face into three areas of interest (AOIs) defined by manual delineation, employing a uniform AOI template for all faces. Consistent with the degree of facial exposure in both static photographs and dynamic video materials, a unified AOI delineation standard was applied, segmenting the face into three distinct regions of interest: the eyes, nose, and mouth. This research used two eye-tracking experiments to explore differences in cross-modal facilitation effects between self-face and other-facial identity recognition, as well as to examine the role of voice in facial identity recognition patterns across the early and late stages of processing.

On the basis of theories concerning the self-advantage effect and cross-modal integration, this study proposes the following core hypotheses: First, mask occlusion interferes with facial identity recognition. This interference will be greater for others than for oneself. Moreover, as half masks are not commonly worn, we anticipate that facial recognition will be more significantly disrupted under half-mask conditions. Second, voice produces cross-modal facilitation for facial recognition, with this facilitation being stronger under mask-obscured conditions than under no-masked conditions, and more pronounced when individuals recognize others’ faces than when they recognize self-faces. Additionally, with respect to the anticipated results of eye-tracking, auditory presentation shortens the early gaze duration and reduces the fixation counts and total fixation duration. Finally, we predict that the facilitation effect of voice on dynamic facial recognition (Experiment 2) will be more pronounced than that on static facial recognition (Experiment 1) owing to the temporal synchrony of dynamic audiovisual information.

## 2. Experiment 1

### 2.1. Methods

#### 2.1.1. Participants

G*Power 3.1.9.2 was used to calculate the minimum sample size required for the experiment (Faul et al., 2007). This effect size analysis employs a conservative design to ensure effective detection of the impact of the core variable, mask-wearing. The calculations utilized a large effect size parameter (η_p_^2^ = 0.16), derived from Kollenda and De Haas (2022). Specifically, the interaction effect size between familiarity (familiar, unfamiliar) and facial recognition scores on the mask condition (mask, no mask) in Experiment 4 was η_p_^2^ = 0.16. This design anticipates detecting an interaction between mask wearing and identity recognition in our experiments. The probability of Type I error (α error probability) was set to 0.05, the statistical power (1 − β error probability) was set to 0.9, and the calculated minimum sample size was 16. To avoid an insufficient sample size due to missing eye-tracking data, 35 participants were recruited from among university students. A total of three participants were excluded. Specifically, one participant’s data were excluded because they were not recorded, and another two participants were excluded because they did not achieve the 90% accuracy criterion. The actual sample size was 32 (9 males and 23 females), ranging from 19–28 years. All the participants were right-handed with normal hearing and either normal or corrected vision, and no color blindness or color weakness. The participants were paid 40 CNY as remuneration at the end of the experiment.

#### 2.1.2. Apparatus and Stimuli

An EyeLink 1000 plus infrared reflectance system eye-tracking recorder (SR Research, Mississauga, ON, Canada) was used to record eye-tracking data from the participants’ left eyes, with a sampling frequency of 1000 Hz. The facial stimuli were presented on a Dell LCD monitor, model P1914SF, with a visual screen size of 19 inches and a screen resolution of 1024 × 768 pixels. The refresh rate was 75 Hz, and the participants were located 75 cm from the computer screen. The experimental program was prepared via MATLAB 2016a software and Pychtoolbox, Eyeslink Toolbox 3.0.12.

**Figure 1:**
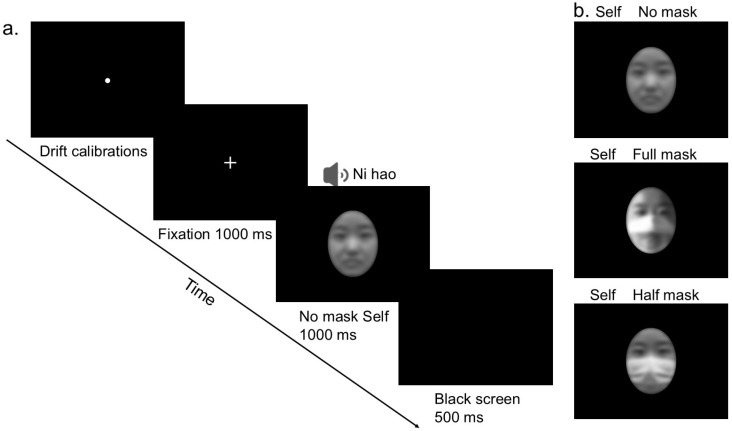
Overview of the procedure in Experiment 1. In panel (**a**), drift correction was applied, followed by the presentation of a central fixation point. Following the fixation point for 1000 ms, a stimulus screen was displayed, asking the participants to judge whether the experimental stimulus represented themselves or another person. The stimulus is exemplified by a self-face without a mask combined with an auditory condition. (**b**). Examples of different types of masks under self-conditions are displayed. To protect the privacy of participants, faces are blurred via Gaussian blurring. The procedure of Experiment 2 was identical to that of Experiment 1, with the only difference being that the experimental stimuli were dynamic faces.

Facial stimuli comprise experimental materials required for prerecorded experiments involving three mask types: participant facial images (no mask, full-mask, half-mask), along with unfamiliar facial images matched to participants’ genders. The full-mask obscures the mouth and nose regions of the face, whereas the half-mask is transparent, obscuring only the participants’ nose (see Figure 1b). Prior to the commencement of the experiment, photographs of the participants’ bare faces wearing a full mask, half mask, and no mask were taken. All the photographs were taken against a pure white background. These photographs were subsequently processed via Adobe Photoshop 2022, with the saturation adjusted to −100. The eyebrows and forehead were retained during cropping, and extraneous stimuli such as hair and ears were removed, keeping the nose of the face in the center of the screen at all times. The cropped stimulus size was presented at a viewing angle of 10° × 14°.

The voice stimuli consisted of ‘Ni Hao’ voices from participants wearing three mask types (no mask, full-mask, half-mask) and from strangers matched to the participants’ gender. These data were produced and processed via Adobe Premiere Pro 2022 and Adobe Audition 2022 at a sampling rate of 44,100 Hz, with a volume of 65 dB and a duration of 1000 ms, including 100 ms of fade-in and fade-out times. Presented through speakers located on the back side of the screen.

#### 2.1.3. Procedure and Design

The experiment utilized a 3 (stimulus type: voice, face, faces + voices) × 2 (identity: self, other) × 3 (mask type: no mask, full-mask, half-mask) three-factor within-participants experimental design.

To ensure that the participants could answer the questions accurately, a practice experiment was conducted before the formal experiment, and the accuracy rate reached 90% (Rinck et al., 2022). To minimize head movements to obtain more accurate eye-tracking data, the participants were instructed to perform a nine-point calibration with the forehead resting against the forehead and the chin resting on the chin rest before starting the experiment. After the calibration was completed, the experiment was started.

The specific experimental procedure is shown in Figure 1a, where a drift calibration point is presented in the center of the screen before each trial presentation. After successful calibration, the calibration point disappeared, and a 1000 ms cross-fixation point (155.2 cd/m^2^, 0.5° × 0.5°) was presented in the center of the screen. After the fixation point disappeared, a 1000 ms stimulus screen was presented in the center of the screen. The stimulus was divided into three cases: face, voice, and faces + voices of the same participant, followed by a 500 ms black screen. The participants were instructed to rapidly categorize each stimulus as “self” or “others” within a 1500 ms response window (including both stimulus presentation and subsequent blank screen periods). Responses were made via a two-alternative forced-choice paradigm with the ‘J’ and ‘F’ keys, where key assignment to response categories was counterbalanced across participants to control for handedness bias. If the participants responded within 1000 ms they skipped the black screen and went directly to the trial, and if they did not respond by pressing a key after 1500 ms, they would go to the next trial.

The formal experiment was divided into 6 blocks. In each block, the 18 conditions were each presented 10 trials in a randomized order, resulting in 180 trials per block and 1080 trials in total. The duration of the entire experiment was 60 min.

#### 2.1.4. Data Analysis

Response time refers to the time interval between the presentation of the stimulus screen and the participant’s response. The Shapiro–Wilk test (S–W test) results indicate that *ps* > 0.05, demonstrating that the experimental data approximately follow a normal distribution. Consequently, test data with reaction times exceeding three standard deviations were excluded (Huber, 2018). The final number of rejected trials was 1.77% of the total number of trials. Two participants who were less than 90% correct in the formal experiment were excluded. A 3 (stimulus type: voice, face, faces + voices) × 2 (identity information: self, other) × 3 (mask type: no mask, full-mask, half-mask) repeated-measures ANOVA was performed on the reaction times of all trials with correct responses.

To further explore the promotional role of voice in different stages of facial identity recognition, this study selected one early and two late eye-tracking indicators (Hardardottir et al., 2020; Salehi Fadardi et al., 2022; Zhang et al., 2022). The early eye-tracking indicator is gaze duration, which refers to the gaze duration between the first gaze at an area of interest and the first departure from that area of interest. In facial processing research, gaze duration is often regarded as an indicator of early, automated information extraction from specific facial regions (Henderson & Hollingworth, 1999; Yan et al., 2013). The late eye-tracking indicators are (1) the fixation count, which refers to the number of times the gaze falls on a fixation point within the region of interest, and (2) the total fixation time, which refers to the sum of the fixation times for all fixation points within the region of interest. These two indicators reflect the late stages of facial identity recognition processing; the higher the fixation count and the longer the total fixation time are, the greater the attention given to the face (Yan et al., 2013). For the eye-movement metrics, it was not meaningful to analyse the voice condition; so, the repeated-measures ANOVA of 3 (areas of interest: eyes, nose, mouth) × 2 (stimulus type: face, faces + voices) × 2 (identity: self, other) × 3 (mask type: no mask, full-mask, half-mask) was performed for all eye-movement metrics. Bonferroni correction was applied in the simple effect analyses.

### 2.2. Results

#### 2.2.1. Behavioral Data

**Figure 2:**
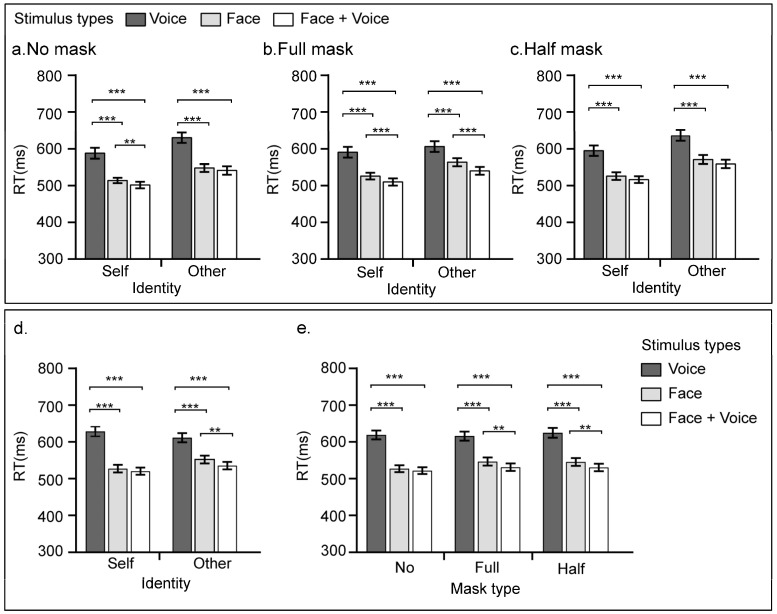
Behavioral results of this study. Panels (**a**–**c**) show the average reaction time (RT, ms) for two types of identity information (self, other) and different types of stimuli (face, faces + voices) under three masking conditions (no mask, full-mask, and half-mask) in Experiment 1. Panels (**d**,**e**) represent the mean RT for different identity information and the three stimulus conditions in Experiment 2. The dark gray bars represent the average RT for face stimuli under each masking condition, the light gray bars represent the average RT for voice stimuli under each masking condition, and the white bars represent the average RT for faces + voices stimuli under each masking condition. The error bars indicate the 95% confidence intervals. All significant differences are indicated with an asterisk (** *p* < 0.01, *** *p* < 0.001).

The descriptive statistics of the behavioral results of Experiment 1 are shown in Table 1. A 2 × 3 × 3 repeated measures ANOVA was performed on the response times, and the results are shown in Figure 2. A notable main effect of stimulus type was observed [*F*(2, 62) = 72.71, *p* < 0.001, η_p_^2^ = 0.70]. The post hoc test results indicate that participants responded significantly slower when they saw voice alone than when they saw faces or faces + voices (*ps* < 0.001). Moreover, the response times for faces were also significantly slower than those for faces presented with voice (*p* < 0.001). A significant main effect of identity was found [*F*(1, 31) = 74.89, *p* < 0.001, η_p_^2^ = 0.70]. The post hoc test results indicate that quicker response times were observed for self-identities than for other identities (*p* < 0.001). Additionally, the main effect of mask presence was significant [*F*(2, 62) = 15.85, *p* < 0.001, η_p_^2^ = 0.34]. According to the post hoc tests, no significant difference in response times was found between conditions with no mask and those with a full-mask (*p* = 1). However, the reaction times were significantly faster in the no-mask and full-mask conditions than in the half-mask condition (*ps* < 0.001).

As shown in Figure 2a–c, there was a significant three-way interaction effect between stimulus type, mask type, and identity information [*F*(4, 124) = 5.05, *p* < 0.001, η_p_^2^ = 0.14]. Simple effect analysis revealed that regardless of whether the identity information was self or other, the response times for faces + voices were significantly faster than those for faces and voices (*ps* < 0.001), and the response times for faces were significantly faster than those for voices (*p* < 0.001). In the no-mask condition, however, the interaction between stimulus type and identity information was not significant [*F*(2, 62) = 0.67, *p* = 0.515]. In the half-mask condition, the interaction effect between stimulus type and identity information was not significant [*F*(2, 62) = 0.14, *p* = 0.866]. The interaction effect between stimulus type and identity information was significant in the full-mask condition [*F*(2, 62) = 80.98, *p* < 0.001, η_p_^2^ = 0.23]. Further simple effect analyses revealed that response times were significantly faster when identity information was self than when it was other (*ps* < 0.05) for all three stimulus types.

#### 2.2.2. Eye-Tracking Data

A total of 0.98% of the trials with an average total fixation duration of 70 ms or less or 900 ms or more were rejected.

Gaze duration. The descriptive statistics for gaze duration are shown in Table 2. The main effect of the area of interest was significant [*F*(2, 62) = 113.63, *p* < 0.001, η_p_^2^ = 0.79]. The post hoc test results indicate that the gaze duration in the mouth area of interest was significantly shorter than that in the eyes and nose areas of interest (*ps* ≤ 0.001), whereas the gaze duration in the nose area of interest was significantly longer than that in the eyes area of interest (*p* < 0.001). The main effect of stimulus type was significant [*F*(1, 31) = 10.87, *p* = 0.002, η_p_^2^ = 0.26]. The post hoc test results indicate that the gaze duration for faces was significantly longer than that for faces + voices (*p* = 0.002). The main effect of mask type was significant [*F*(2, 62) = 5.01, *p* = 0.010, η_p_^2^ = 0.14]. The post hoc test results indicate that the gaze duration for no mask was significantly shorter than that for a half-mask (*p =* 0.028), whereas there was no significant difference in gaze duration between the full-mask and no mask compared with the half-mask (*ps* ≥ 0.05). The main effect of identity information was significant [*F*(1, 31) = 46.73, *p* < 0.001, η_p_^2^ = 0.60]. The post hoc test results indicate that the gaze duration for oneself was significantly shorter than that for others (*p* < 0.001).

**Figure 3:**
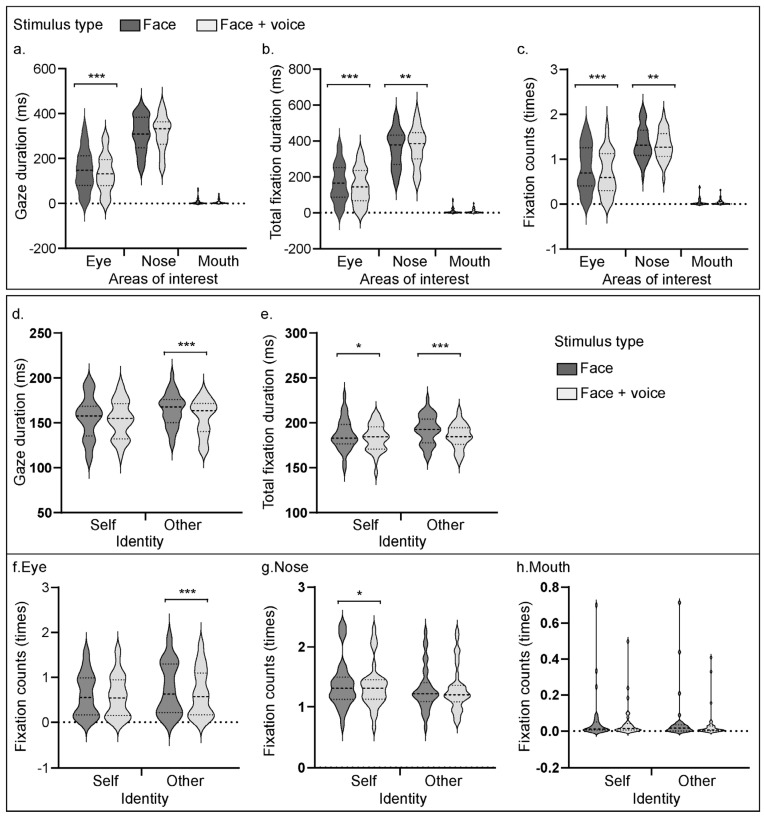
Eye-tracking results. Panels (**a**–**c**) show the eye-tracking results for Experiment 1. Panels (**d**–**h**) show the eye-tracking results for Experiment 2. The thin dashed lines indicate quartiles, whereas the thick dashed lines denote the median. Panel (**a**) shows the gaze duration (ms) for two stimulus types (face, faces + voices) across different areas of interest (AOIs: eyes, nose, mouth) in Experiment 1. The dark gray violin plots represent the facial condition, whereas the light gray violin plots represent the faces + voices condition. Panel (**b**) shows the total fixation duration (ms) for both stimulus types across different AOIs. Panel (**c**) shows the fixation duration (times) for both stimulus types across different AOIs. Panel (**d**) illustrates the differences in gaze duration across stimulus types under the two identity conditions (self, other). Panel (**e**) shows the differences in total fixation duration across stimulus types under the two identity conditions. Panels (**f**–**h**) show the differences in fixation counts across different AOIs for the two identity conditions. All significant differences are indicated with an asterisk (* *p* < 0.05, ** *p* < 0.01, *** *p* < 0.001).

As shown in Figure 3a, the interaction effect between the area of interest and the type of stimulus was significant [*F*(2, 62) = 7.28, *p* = 0.001, η_p_^2^ *=* 0.19]. The results of the simple effect analysis indicate that in the eye area of interest, the gaze duration of the face was significantly longer than that of the faces + voices (*p* < 0.001). In the nose and mouth areas of interest, there was no significant difference between the gaze duration of the face and that of the faces + voices (*ps* ≥ 0.05).

The three-way interaction effect between the area of interest, mask type, and identity information was significant [*F*(4, 124) = 7.04, *p* < 0.001, η_p_^2^ = 0.19]. The results of the simple effect analysis indicate that in the eye area of interest, under no-mask conditions, there was no significant difference between self-gaze duration and others’ gaze duration (*p* = 0.249). Under the full-mask and half-mask conditions, self-gaze duration was significantly shorter than others’ gaze duration (*ps* ≤ 0.001). In the mouth area of interest, under no-mask conditions, there was no significant difference between self-gaze duration and others’ gaze duration (*p* = 0.248). Under the full-mask and half-mask conditions, the gaze duration of the self was significantly shorter than that of the others (*ps* ≤ 0.05). In the area of interest of the nose, under the condition of no mask, the gaze duration of the self was significantly shorter than that of the others (*p* = 0.007). Under the full-mask and half-mask conditions, there was no significant difference between the gaze duration of the self and others (*ps* ≥ 0.05).

There was a significant interaction effect between areas of interest and mask type [*F*(4, 124) = 10.16, *p* < 0.001, η_p_^2^ = 0.25]. There was a significant interaction effect between areas of interest and identity information [*F*(2, 62) = 7.46, *p* = 0.001, η_p_^2^ = 0.19].

No other interactions were significant [*Fs* < 2, *ps* > 0.05].

Total fixation duration. The descriptive statistics for total fixation duration are shown in Table 2. There was a significant main effect of areas of interest [*F*(2, 62) = 98.64, *p* < 0.001, η_p_^2^ = 0.76]. The post hoc test results indicate a significantly greater total fixation duration for the nose than for the eyes and mouth (*ps* < 0.001) and a significantly greater total fixation duration for the eyes than for the mouth (*p* < 0.001). There was a significant main effect of identity information [*F*(1, 31) = 50.13, *p* < 0.001, η_p_^2^ = 0.62]. The post hoc test results indicate significantly less total fixation duration for oneself than for others (*p* < 0.001). There was a significant main effect of mask type [*F*(2, 62) = 10.65, *p* < 0.001, η_p_^2^ = 0.26]. The post hoc test results indicate that the total fixation duration was significantly lower for no mask than for half-masks (*p* < 0.001), and significantly lower for full-masks than for half-masks (*p* = 0.011); however, there was no significant difference between the total fixation duration of no mask and that of full-masks (*p* = 0.859).

As shown in Figure 3b, the interaction effect between the areas of interest and stimulus type was significant [*F*(2, 62) = 14.51, *p* < 0.001, η_p_^2^ = 0.32]. The results of the simple effect analysis indicate that in the eye areas of interest, the total fixation duration for faces was significantly greater than that for faces + voices (*p* < 0.001); in the nose areas of interest, the total fixation duration for faces was significantly less than that for faces + voices (*p* = 0.004); and in the mouth areas of interest, there was no significant difference in total fixation duration for faces or for faces + voices (*p* = 0.178).

As shown in Figure 3c, the interaction effect between mask type and stimulus type was significant [*F*(2, 62) = 4.64, *p* = 0.013, η_p_^2^ = 0.13]. A simple effect analysis indicated that there was no significant difference between the total fixation duration for faces and faces + voices in the no mask condition (*p* = 0.691); the total fixation duration for faces and faces + voices in the full-mask condition was not significantly different (*p* = 0.648); and the total fixation duration for faces was significantly greater than that for faces + voices in the half-mask condition (*p* < 0.001).

The three-way interaction effect of areas of interest, mask type, and identity was significant [*F*(4, 124) = 5.39, *p* < 0.001, η_p_^2^ = 0.15]. The simple effect analysis indicated that under the eye areas of interest, the total fixation duration was significantly shorter for oneself than for others in the no-mask condition (*p* < 0.001), and the difference between the total fixation duration for oneself and others was not significant in the full-mask and half-mask conditions. In the nose areas of interest, there was a significantly greater total fixation duration for oneself than for others in the no mask condition (*p* = 0.025), a significantly greater total fixation duration for oneself than for others in the full-mask condition (*p* = 0.009), and there was no significant difference between the total fixation duration for oneself and others in the half-mask condition (*p* = 0.107). In the mouth areas of interest, the total fixation duration was significantly greater for oneself than for others in the no mask condition (*p* = 0.047), not significantly different between oneself and others in the full-mask condition (*p* = 0.087), and significantly shorter for oneself than for others in the half-mask condition (*p* = 0.017).

The interaction effect between areas of interest and identity was significant [*F*(2, 62) = 7.60, *p* = 0.001, η_p_^2^ = 0.20]. The interaction effect between the areas of interest and mask type was significant [*F*(4, 124) = 11.93, *p* < 0.001, η_p_^2^ = 0.28].

No other interactions were significant [*Fs* < 2, *ps* > 0.05].

Fixation counts. Descriptive statistics for the eye-tracking indicators are shown in Table 2. The results of the four-factor repeated measures analysis of variance are shown in Figure 3. There was a significant main effect of area of interest [*F*(2, 62) = 88.74, *p* < 0.001, η_p_^2^ = 0.74]. The post hoc test results indicate that the fixation counts of the eyes and mouth areas of interest were significantly lower than those of the nose areas of interest (*ps* < 0.001), and that the fixation counts of the eye areas of interest were significantly greater than those of the mouth areas of interest (*p* < 0.001). There was a significant main effect of stimulus type [*F*(1, 31) = 38.35, *p* < 0.001, η_p_^2^ = 0.55]. The post hoc test results indicate that the fixation counts for faces was significantly greater than the fixation counts for faces + voices (*p* < 0.001). There was a significant main effect of mask type [*F*(2, 62) = 19.50, *p* < 0.001, η_p_^2^ = 0.39]. The post hoc test results indicate that the fixation counts of no mask was significantly lower than the fixation counts of both full and half-masks (*ps* < 0.001) and that the fixation counts of the full-mask were significantly greater than those of the half-mask (*p* = 0.007). There was a significant main effect of identity [*F*(1, 31) = 24.76, *p* < 0.001, η_p_^2^ = 0.44]. The post hoc test results indicate that significantly fewer fixation counts for self than for others (*p* < 0.001).

As shown in Figure 3c, the interaction effect between areas of interest and stimulus type was significant [*F*(2, 62) = 9.50, *p* < 0.001, η_p_^2^ = 0.24]. The simple effect analysis indicated that the fixation counts for faces were significantly greater than those for faces + voices (*p* < 0.001) under the eye areas of interest, and the fixation counts for faces were significantly greater than those for faces + voices (*p* = 0.004) under the nose areas of interest. Under the mouth areas of interest, there was no significant difference between the fixation counts of sessions for the face and those for faces + voices (*p* = 0.251).

The interaction effect of mask type and stimulus type was marginally significant [*F*(2, 62) = 2.91, *p* = 0.062, η_p_^2^ = 0.07]. The simple effect analysis indicated that for all three types of mask type variables, the fixation counts for faces were significantly greater than those for faces + voices (*ps* < 0.001).

The three-way interaction effect between areas of interest, mask type, and identity was significant [*F*(4, 124) = 4.75, *p* = 0.001, η_p_^2^ = 0.13]. A simple effect analysis revealed that in terms of the area of interest of the eyes, there was no significant difference between the fixation counts of the self and others under the condition of no mask (*p* = 0.309), whereas under the conditions of half-mask and full-mask, the fixation counts of the self were significantly lower than those of the others (*ps* < 0.001). In the area of interest around the nose and mouth, there was no significant difference between the time spent looking at oneself and that spent looking at others under any mask type (*ps* > 0.05).

No other interactions were significant [*Fs* < 2, *ps* > 0.05].

**Figure 4:**
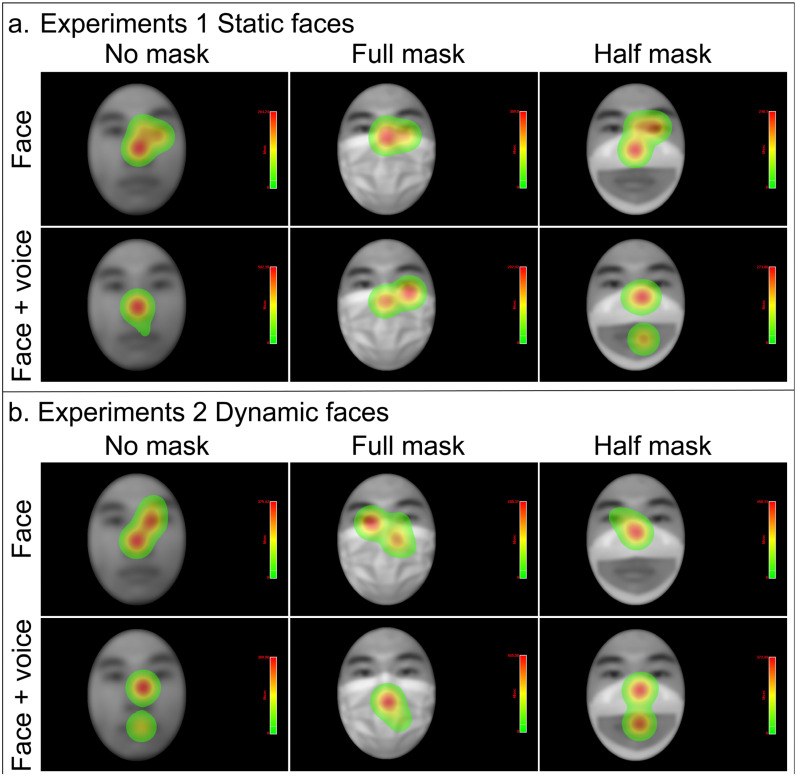
Heatmap of Experiments 1 and 2. Panel (**a**) shows the heatmap for Experiment 1, and Panel (**b**) shows the heatmap for Experiment 2. These heatmaps illustrate the total fixation duration under different stimulus types (face, faces + voices) and mask types (no mask, full-mask, half-mask), where colors represent the magnitude of the total fixation duration. The redder the color is, the longer the total fixation duration is. To protect the privacy of participants, faces are blurred via Gaussian blurring.

Figure 4a presents the heatmap for Experiment 1, allowing the differences in how stimulus conditions and mask types affect total fixation duration to be observed more intuitively. Across the three mask types, fixated areas during facial-identity identification are concentrated mainly on the eyes and nose. The total fixation duration in the face condition was longer than that in the face + voice condition, and the total fixation duration in the half-mask condition was significantly longer than that in the no-mask or full-mask condition.

The results of Experiment 1 indicate that mask occlusion significantly prolonged reaction times in static facial recognition tasks, supporting the hypothesis that occlusion impairs identity recognition. This study identified a stable self-advantage effect, in which self-faces were recognized more rapidly than others were, which is consistent with the hypothesis that “self-faces possess a processing advantage”. However, no self-advantage effect was observed under the voice condition. Furthermore, the addition of voice significantly enhanced facial recognition efficiency, particularly when faces were masked. Eye-tracking data revealed that voice significantly shortened gaze duration, indicating its facilitative role during the early stages of facial recognition. This facilitation effect was not observed during the later stages of facial recognition. After the face was obscured by the mask, participants reduced fixation on the mouth region, instead of focusing more on the eye and nose areas, suggesting that occlusion triggered a localized processing strategy. Under occluded conditions, both the fixation counts and total fixation duration were significantly greater for other people’s faces than for one’s own face, demonstrating greater efficiency in self-processing and supporting the cognitive resource hypothesis of the self-advantage effect.

## 3. Experiment 2

### 3.1. Methods

#### 3.1.1. Participants

Consistent with Experiment 1, the minimum sample size required for the experiment was calculated via the G*Power toolbox 3.1.9.2 (Faul et al., 2007). The calculation yielded a minimum sample size of 16. To avoid a shortage in sample size due to missing eye-tracking data, 33 university students who had not participated in Experiment 1 were recruited. One participant was excluded because the accuracy rate did not reach 90%, resulting in an actual sample size of 32 individuals (11 males and 21 females) aged between 19 and 28 years. All the participants were right-handed, with normal hearing, vision, or corrected vision, and without color blindness or color weakness. The participants received appropriate compensation upon completion of the experiment.

#### 3.1.2. Apparatus and Stimuli

The apparatus and stimuli for Experiment 2 were the same as those used in Experiment 1, with the following exceptions. First, the facial stimuli used in Experiment 2 were prerecorded as dynamic faces with a video duration of 1000 ms. Second, the voice stimuli were collected through recorded videos and synchronized with the facial information.

#### 3.1.3. Procedure and Design

A 3 (stimulus type: voice, face, faces + voices) × 2 (identity information: self, other) × 3 (mask type: no mask, full-mask, half-mask) three-factor within-participants experimental design was used. The specific flow of the experiment was identical to that of Experiment 1. The formal experiment consisted of six groups, each with a stimulus condition presentation time of 1000 ms, and each group had 180 trials for a total of 1080 trials. The formal experiment was preceded by practice trials. The participants were allowed to rest between groups, and the entire experiment lasted approximately 60 min.

#### 3.1.4. Data Analysis

The data analysis was the same as that in Experiment 1.

### 3.2. Results

#### 3.2.1. Behavioral Data

The data exclusion criteria were the same as those in Experiment 2, and 1.59% of the total number of trials were ultimately excluded.

The descriptive statistics of the behavioral results of Experiment 2 are shown in Table 3. A 2 × 3 × 3 repeated-measures ANOVA was performed on the response times, with a significant main effect for the type of stimulus [*F*(2, 62) = 168.44, *p* < 0.001, η_p_^2^ = 0.85]. The post hoc test results indicate that the RTs were significantly longer for voice stimuli than for face stimuli (*p* < 0.001). Additionally, voice RTs were significantly slower than those for combined face and voice stimuli (*p* < 0.001). The face condition also elicited slower RTs than did the faces + voices condition did (*p* = 0.003). Moreover, a significant main effect was observed for mask presence [*F*(2, 62) = 11.27, *p* < 0.001, η_p_^2^ = 0.27]. The post hoc test results indicate that the RTs in the no-mask conditions were significantly faster than those in the full-mask conditions were (*p* = 0.004), and the no-mask conditions presented significantly faster RTs than did the half-mask conditions (*p* < 0.001). No significant difference in RT emerged between the full-mask and half-mask conditions (*p* = 0.963).

The interaction effect between stimulus type and identity was notably significant [*F*(2, 62) = 20.57, *p* < 0.001, η_p_^2^ = 0.40], as shown in Figure 2d. The results of the simple effect analysis indicate that for the self-condition, the RTs for the voice alone condition were markedly slower than those for the face (*p* < 0.001) and the combined face and voice stimuli (*p* < 0.001). Although the RTs for faces were longer than those for faces + voices, this difference was not statistically significant (*p* = 0.062). In contrast, for stimuli depicting others, voice-alone stimuli also prompted slower RTs than face (*p* < 0.001) and face + voice conditions did (*p* < 0.001). Here, the face condition led to significantly slower RTs than did the faces + voices condition (*p* = 0.002).

Additionally, the interaction effect between stimulus type and mask type was significant [*F*(4, 124) = 5.1, *p* < 0.001, η_p_^2^ = 0.14], as shown in Figure 2e. The results of the simple effect analysis indicate that in the conditions with no mask, voice stimuli alone resulted in slower RTs than did face stimuli (*p* < 0.001) and faces + voices stimuli did (*p* < 0.001), although no significant RT differences were found between the latter two conditions (*p* = 0.317). Within the full-mask condition, voice-alone RTs were again slower than those for faces (*p* < 0.001) and faces + voices (*p* < 0.001), and RTs for faces were also slower than those for faces + voices (*p* = 0.004). In the half-mask condition, voice alone elicited slower responses than did face (*p* < 0.001) or faces + voices (*p* < 0.001). In addition, the face condition had slower RTs than did the faces + voices condition did (*p* = 0.002).

No other interactions were significant [*Fs* < 2, *ps >* 0.05].

#### 3.2.2. Eye-Tracking Data

A total of 1.02% of the trials with an average total fixation duration of less than 70 ms or more than 900 ms were excluded. ANOVA with repeated measures of 3 (areas of interest: nose, eyes, mouth) × 2 (stimulus type: face, faces + voices) × 3 (mask type: no mask, half-mask, full-mask) × 2 (identity: self, other) was performed.

Gaze duration. The descriptive statistics for gaze duration are shown in Table 4. The main effect of the area of interest was significant [*F*(2, 62) = 116.64, *p* < 0.001, η_p_^2^ = 0.79]. The post hoc test results indicate that the gaze duration of the mouth area of interest was shorter than that of the eyes and nose areas of interest (*ps* < 0.001) and that the gaze duration of the eye area of interest was significantly shorter than that of the nose area of interest (*p* < 0.001). The main effect of stimulus type was significant [*F*(1, 31) = 16.86, *p* < 0.001, η_p_^2^ = 0.35]. The post hoc test results indicate that the gaze duration for faces was significantly longer than the gaze duration for faces + voices (*p* < 0.001). The main effect of mask type was significant [*F*(2, 62) = 12.93, *p* < 0.001, η_p_^2^ = 0.29]. The post hoc test results indicate that the gaze duration was significantly shorter for the no mask condition than for the full-mask and half-mask conditions (*ps* ≤ 0.05), and there was no significant difference in gaze duration between the full-mask and half-mask conditions (*p* = 0.170). The main effect of identity information was significant [*F*(1, 31) = 9.74, *p* = 0.004, η_p_^2^ = 0.24]. The post hoc test results indicate that the gaze duration for oneself was significantly shorter than the gaze duration for others (*p* = 0.004).

There was a significant interaction effect between the area of interest and mask type [*F*(4, 124) = 11.52, *p* < 0.001, η_p_^2^ = 0.27]. The results of the simple effect analysis indicate that in the eye area of interest, the gaze duration without a mask was significantly shorter than that with a full-mask and half-mask (*p* < 0.001), and the gaze duration with a full-mask was significantly longer than that with a half-mask (*p* = 0.002). There was no significant difference in gaze duration among the three types of masks in the nose and mouth areas of interest (*ps* ≥ 0.05).

There was a significant interaction effect between the area of interest and identity information [*F*(2, 62) = 7.75, *p* < 0.001, η_p_^2^ = 0.20], as shown in Figure 2d. The results of the simple effect analysis indicate that in the eye area of interest, the gaze duration on the self was significantly shorter than that on others (*p* < 0.001); in the mouth and nose areas of interest, there was no significant difference between the gaze duration on the self and others (*ps* ≥ 0.05).

The interaction effect between stimulus type and identity information was significant [*F*(1, 31) = 11.00, *p* = 0.002, η_p_^2^ = 0.26]. The results of the simple effect analysis indicate that under the self-condition, there was no significant difference in gaze duration between faces and faces + voices (*p* = 0.280); under the other condition, gaze duration for the face was significantly longer than gaze duration for the faces + voices (*p* < 0.001). On the other hand, under the face condition, the gaze duration of the self was significantly shorter than that of others (*p* < 0.001), and under the faces + voices condition, the difference between the gaze duration of the self and others was not significant (*p* = 0.342).

No other interactions were significant [*Fs* < 2, *ps* > 0.05].

Total fixation duration. The descriptive statistics for total fixation duration are shown in Table 4. The areas of interest revealed a significant main effect [*F*(2, 62) = 91.05, *p* < 0.001, η_p_^2^ = 0.75]. The post hoc test results indicate that the participants fixated the longest time on the nose, which was substantially longer than fixations on the eyes and the mouth, with both comparisons yielding highly significant differences (*ps* < 0.001). Fixation on the eyes also significantly exceeded that on the mouth (*p* < 0.001). With respect to stimulus type, a significant main effect emerged [*F*(1, 31) = 11.61, *p* = 0.002, η_p_^2^ = 0.27]. The post hoc test results indicate that faces viewed in isolation attracted a longer total fixation duration than did faces + voices did, and this difference was statistically significant (*p* = 0.002). The effect of identity information was also significant [*F*(1, 31) = 24.02, *p* < 0.001, η_p_^2^ = 0.44]. The post hoc test results indicate that self-faces resulted in a shorter total fixation duration than did the faces of others did, with the difference being highly significant (*p* < 0.001). The type of mask worn by the participants had a significant main effect [*F*(2, 62) = 8.87, *p* < 0.001, η_p_^2^ = 0.22]. The post hoc test results indicate that the no mask condition had a shorter total fixation duration than did the full-mask (*p* = 0.014) and half-mask (*p* = 0.001) conditions. Nevertheless, no significant difference was found between the total fixation duration for no mask faces and those with full masks (*p* = 0.608).

The interaction effect between the areas of interest and stimulus type was found to be significant [*F*(2, 62) = 4.93, *p* = 0.010, η_p_^2^ = 0.14]. The results of the simple effect analysis indicate that within the eye area, the total fixation duration was significantly greater for individual faces than for faces accompanied by voices (*p* = 0.004). However, no significant differences in total fixation duration were detected for the nose area between individual faces and faces + voices (*p* = 0.145) or for the mouth area between faces and faces + voices (*p* = 0.080).

Moreover, a significant interaction effect emerged between the areas of interest and the type of mask [*F*(4, 124) = 16.53, *p* < 0.001, η_p_^2^ = 0.35]. The results of the simple effect analysis indicate that for the eye areas of interest, the total fixation duration was significantly shorter for no mask faces than for faces with full-masks (*p* < 0.001) and faces with half-masks (*p* < 0.001). Additionally, the total fixation duration for faces with full-masks was significantly longer than that for faces with half-masks (*p* = 0.002). In the nose areas of interest, the longest total fixation duration was observed for no mask faces, which was significantly longer than that for faces with full-masks (*p* < 0.001) and half-masks (*p* = 0.024). The full-mask also attracted significantly shorter fixations than did the half-mask (*p* = 0.006). The differences between the three mask types are not significant in the mouth areas (*ps* > 0.05).

A significant interaction effect between mask type and stimulus type was found [*F*(2, 62) = 3.95, *p* = 0.024, η_p_^2^ = 0.11]. The results of the simple effect analysis indicate that for the no-mask condition, no significant disparity in total fixation duration was noted between individual faces and faces paired with voices (*p* = 0.362). Similarly, with a full-mask, the total fixation duration did not significantly differ between individual faces and faces + voices (*p* = 0.648). In contrast, under the half-mask condition, individual faces garnered a significantly longer total fixation duration than faces accompanied by voices did (*p* < 0.001).

As shown in Figure 2e, the interaction effect between the areas of interest and identity was significant [*F*(2, 62) = 8.32, *p* < 0.001, η_p_^2^ = 0.21]. The results of the simple effect analysis indicate that within the eye area of interest, the total fixation duration was notably shorter for the self-condition than for the other conditions (*p* < 0.001). No significant difference emerged for the nose area between self and others (*p* = 0.264). Similarly, the total fixation duration for the mouth area did not significantly differ between themselves and the other areas (*p* = 0.844).

The interaction effect between mask type and identity was also significant [*F*(2, 62) = 3.28, *p* = 0.044, η_p_^2^ = 0.10]. The results of the simple effect analysis indicate that participants’ total fixation duration on self-faces was significantly shorter than that of others in all three mask type conditions (*ps* ≤ 0.003). In the self-condition, the total fixation duration was significantly shorter without a mask than with a full mask (*p* = 0.032), whereas no significant difference was detected between the no-mask condition and the half-mask condition (*p* = 0.259). No significant difference was found when the full-mask condition was compared with the half-mask condition (*p* = 1) in the self-condition. For the other conditions, there was no significant difference in total fixation duration between the no-mask and the full-mask conditions (*p* = 0.238). However, the total fixation duration without a mask was significantly shorter than that with a half-mask (*p* < 0.001), with no significant difference noted between the full-mask and the half-mask conditions (*p* = 0.070).

No other interactions were significant [*Fs* < 2, *ps* > 0.05].

Fixation counts. Descriptive statistics for the eye-tracking indicators are shown in Table 4. With respect to the areas of interest, a significant main effect was found [*F*(2, 62) = 81.33, *p* < 0.001, η_p_^2^ = 0.72]. The post hoc test results indicate that in the eye area of interest significantly fewer fixation counts were received on average than in the nose area (*p* < 0.001). Conversely, the eye area attracted significantly greater fixation counts than did the mouth area of interest (*p* < 0.001). Additionally, the nose area was the focus of significantly greater fixation counts than the mouth area of interest was (*p* < 0.001). The type of stimulus also had a significant main effect [*F*(1, 31) = 12.64, *p* < 0.001, η_p_^2^ = 0.29]. The post hoc test results indicate that the face garnered greater fixation counts than did the combined face and voice condition (*p* = 0.001). Furthermore, a significant main effect of mask type was detected [*F*(2, 62) = 40.69, *p* < 0.001, η_p_^2^ = 0.57]. The post hoc test results indicate that the fixation counts in the no-mask condition were significantly lower than those in the full-mask condition (*p* < 0.001) and the half-mask condition (*p* < 0.001). Compared with the half-mask, the full-mask also prompted significantly greater fixation counts (*p* < 0.001).

There was a significant three-way interaction effect between area of interest, stimulus type, and identity information [*F*(2, 62) = 5.655, *p* = 0.006, η_p_^2^ = 0.154]. A simple effect test revealed that in the eye area of interest, as shown in Figure 3f, the difference in fixation counts between the two stimuli of the self was not significant (*p* = 0.357), but when the identity information was that of others, the fixation counts of the faces were significantly greater than those of the faces + voices (*p* < 0.001). In the nose area of interest, the fixation counts for the faces in the self-condition were significantly greater than those for the faces + voices (*p* = 0.05), as shown in Figure 3g, whereas the difference in fixation counts between the two types of stimuli for others was not significant (*p* = 0.931). In the mouth area of interest, the difference in fixation counts between the face and the faces + voices was not significant for either the self or others (*ps* > 0.05), as shown in Figure 3h. On the other hand, in the eye area of interest, whether it was a face or a face + voice, the fixation counts for the self were significantly lower than those for the others (*ps* < 0.05). In the nose area of interest, under the condition of a face as the stimulus type, the fixation counts for the self were significantly greater than those for others (*p* = 0.030). Under the condition of faces + voices, the difference between the fixation counts of the self and others was not significant (*p* = 0.030). In the mouth area of interest, whether it was face or faces + voices, the difference between the fixation counts of the self and others was not significant (*ps* > 0.05).

The interaction effect between the areas of interest and mask type was statistically significant [*F*(4, 124) = 15.38, *p* < 0.001, η_p_^2^ = 0.33]. The results of the simple effect analysis indicate that a significant interaction effect was found between mask type and stimulus type [*F*(2, 62) = 4.01, *p* = 0.023, η_p_^2^ = 0.12].

No other interactions were significant [*Fs* < 4, *ps* > 0.05].

To visually compare the differences between faces and faces + voices stimuli across mask types, heatmaps were generated, as shown in Figure 4b. In the no-mask, face condition, the participants focused primarily on the nose. In the faces + voices condition, both the full-mask and half-mask trials drew attention mainly to the nose and mouth. The total fixation duration was longer in the face condition than in the face + voice condition.

The results of Experiment 2 indicate that in dynamic facial recognition, mask occlusion similarly significantly prolongs reaction times, with a greater impact on others’ faces than on one’s own face, supporting the hypothesis that “occlusion causes greater interference in recognizing others.” The self-advantage effect under dynamic conditions remains stable, which is consistent with the hypothesis. The facilitative effect of the voice is more pronounced under occlusion conditions. Eye-tracking data further indicate that, compared with self-recognition, voice recognition has a greater facilitative effect on recognition of others’ faces. This facilitation emerges during the early stages of facial recognition and persists through to the later stages. This finding indicates that voice enhances information integration efficiency, supporting the hypothesis that task difficulty modulates cross-modal integration. Furthermore, under full-mask conditions, the fixation concentration shifted to the eye region; whereas under half-mask and no-mask conditions, the fixation distribution expanded across the eye, nose, and mouth regions, reflecting holistic processing strategies during dynamic processing.

## 4. General Discussion

This study employed a facial identity recognition paradigm to investigate the impact of voices on identifying static and dynamic faces occluded by masks. Two eye-tracking experiments were conducted to achieve this goal.

### 4.1. The Self-Advantage Effect in Mask-Obscured Facial Recognition

The results of the two experiments consistently revealed that regardless of the type of mask worn, the recognition of self-faces was greater than that of others. This is known as the self-advantage effect (Ota & Nakano, 2021), which allows individuals to acquire a greater amount of information in a shorter amount of time when they recognize their own face (Jublie & Kumar, 2021). Notably, face masks can disrupt holistic processing (Estudillo & Wong, 2024). Like most studies on self-face advantages, for example, research has indicated that self-faces may be processed more featurally than other faces are processed (Lee et al., 2022a), which could plausibly reduce the impact of occlusion on self-face processing. This study compares self faces with stranger faces. Therefore, the observed self-face advantage may partly stem from the high familiarity of self-faces rather than from purely self-specific processing. This interpretation aligns with the findings of Lee et al. (2023).

The eye-tracking results from Experiments 1 and 2 indicate that when self-faces are identified, the distribution of eye-tracking fixation points across different areas of interest is broader than that when other faces are identified. This aligns with the top-down modulation of attention by identity information mentioned in Scheller and Sui’s (2022) study. However, under the condition of voice alone, the self-advantage effect was not observed. According to previous studies, this is because self-voice recognition is more difficult under air conduction conditions (Iannotti et al., 2021). Previous research on the self-advantage effect of voices has also been inconsistent. Some studies have reported a self-advantage for voices (Xu et al., 2013), whereas others have reported no such advantage (Modelska et al., 2019).

In summary, performance in recognizing one’s own face consistently outperformed recognition of others’ faces across different masking conditions, confirming the existence of the self-advantage effect. This advantage likely stems from two sources: first, processing one’s own face may rely more heavily on feature-based local strategies, rendering it less susceptible to the disruptive effects of masks on holistic processing. Second, given that comparison faces are unfamiliar, this advantage may be partly attributed to exceptionally high familiarity with self-face. However, no self-advantage was observed when voice alone was presented.

### 4.2. Facilitating Effect of Voice on Facial Identity Recognition

The inclusion of voices was found to facilitate facial identity recognition in the reaction time results of Experiments 1 and 2. This finding is consistent with previous studies, which have shown that voice can facilitate performance in face search (Zweig et al., 2015) and face categorization tasks (Abbatecola et al., 2021; O’Mahony & Newell, 2012). Across all the experiments and mask types, the RTs for recognizing voice alone were significantly lower than those for recognizing the face alone. This finding indicates that the faster response times observed when the face and voice were presented together were not due to faster voice recognition, but rather because voice recognition facilitated facial identity recognition directly. While the face and voice have their own distinct processing brain regions, the occipital lobe and the temporal lobe, there are parts of our brain called the right posterior superior temporal sulcus that bind the two as one (Davies-Thompson et al., 2019). Specifically, this binding mechanism appears to be an automatization process prior to attention (Föcker et al., 2011). Therefore, auditory input can influence visual processing in cross-modal tasks and may facilitate task performance. This effect may stem from information redundancy or information integration, although neurological evidence for audiovisual integration is lacking. Consequently, voices can influence visual processing during cross-modality tasks.

The present study also revealed that voices contributed more to the facilitation of mask-occluded faces. One possible explanation for this result is that, when mask-occluded faces are recognized, participants tend to use a local processing strategy (Hsiao & Cottrell, 2008), whereas they tend to use a holistic processing strategy for no mask faces (Maurer et al., 2002; Richler et al., 2011). In other words, in the mask-occluded condition there is less visual information, and when there is less visual information the auditory information becomes more helpful. Although voice facilitates facial identity recognition regardless of whether a mask is worn, voice facilitates the recognition of masked faces more. This finding suggests that the facilitation of voice increases when a local processing strategy is used for facial identity recognition and that this facilitation differs between the two identities. The results of the fixation counts and total fixation duration indicated that the facilitation effect on others’ static faces was greater than that on self-faces. This finding is consistent with previous research: voices matching facial identities enhance facial recognition (Schweinberger et al., 2007, 2011). That is, voice input significantly suppresses mask interference for other people’s faces compared with self-faces, which is consistent with existing research (Iannotti et al., 2021). This is in line with the claim that when task difficulty increases, the facilitation between different senses also increases (Regenbogen et al., 2018), which is referred to as the inverse effect.

Another possible explanation is that voice facilitates the recognition of difficult faces more. The reaction time results revealed that recognition of no-mask faces was easier than recognition of mask-occluded faces. The analysis of the two eye-movement indices revealed that the facilitation of voices on the no mask face was reflected only in the reduction in the fixation counts, whereas the total fixation duration was not significantly reduced. However, the facilitation of voices on the half-masked face was reflected not only in the reduction in the fixation counts, but also in the shortening of the total fixation duration. This suggests that voices facilitate the recognition of faces of different levels of difficulty to different degrees, which is consistent with the inverse ratio effect mentioned earlier (Regenbogen et al., 2018). Compared with the recognition of no mask faces, the presentation of voices improved the processing efficiency of facial identity recognition to a greater extent when recognition of difficult masks occluded faces.

In addition, the comparison of heatmaps revealed that participants focused more on the mouth AOI when viewing dynamic faces than when viewing static faces. This result is consistent with Liu et al.’s finding that dynamic faces receive more attention (Liu et al., 2016). Common coding theory proposes that perceiving an action will activate the same motor plans as those involved in actually performing the action, thereby activating language motor activities related to words in the “mental lexicon” (Tye-Murray et al., 2013). According to common coding theory, compared with static faces, dynamic half-mask faces activate the participants’ “mental lexicon”, allowing them to gather more information about the mouth and thus improving judgment speed.

In conclusion, our results indicate that mask occlusion has a greater effect on the recognition of other people’s faces than on that of self-faces does, confirming the existence of a self-advantage effect. In addition, voice attenuates the interference of mask occlusion on facial identity recognition, and the facilitation effect of voice is greater when a local processing strategy is used for facial identity recognition. Finally, the effect of masks on facial identity recognition is based on a change in processing strategy to localized processing, and the facilitation of voice recognition for masked facial identity recognition is also shaped by acting on facial eye-tracking patterns.

### 4.3. Effect of Masking on Facial Identity Recognition

In both experiments, longer reaction times, an increase in the fixation counts and an increase in the total fixation duration were found when mask-occluded faces were recognized, suggesting that mask occlusion affects our identity recognition of faces. There are several possible reasons for this: first, masking may have altered processing during facial identity recognition. This finding is consistent with the findings of previous research (Estudillo & Wong, 2024). The results of Experiment 1 indicate that when faces without masks and with full- or half-masks are recognized, participants use a fixation strategy of focusing on the nose. The nose serves as the primary fixation point in facial recognition because it occupies the “geographical center” of facial information (Hsiao & Cottrell, 2008). This position maximizes the integration of key feature information within a single or few fixations while aligning with the resolution distribution characteristics of the human visual system. Consequently, it represents an eye-tracking strategy optimized for perceptual and cognitive efficiency (Hsiao & Cottrell, 2008; Peterson & Eckstein, 2012).

As seen in the findings of Experiment 2, the fixation counts and total fixation duration in the nose region significantly exceeded those in the eyes and mouth regions when the participants recognized the face with the full-mask (Hsiao & Cottrell, 2008). When participants perform facial identity recognition, a strategy that focuses on a single site is more likely to assume that they are performing localized processing, whereas a strategy in which they fix at multiple sites is assumed to be holistic processing (Bartlett et al., 2003; Kelly et al., 2010). This finding is consistent with the conjecture of Freud et al. (2020) that mask occlusion causes participants to perform localized processing. However, the fixation counts and total fixation duration in the mouth region increased for the recognition of no mask as well as the half-masked dynamic faces, probably because the mouth under the dynamic faces attracted more attention and thus activated our responsive visual representations in the brain, which is consistent with the findings of a previous study by Tye-Murray et al. (2013).

Our results suggest that participants prefer a holistic processing strategy when recognizing static faces and a localized processing strategy when recognizing dynamic faces. In the full-mask condition, mask-occlusion of the lower part of the face forced the participants to change from holistic processing to localized processing. The participants could not acquire enough information to recognize the face, resulting in a longer response time for facial identity recognition. Second, when wearing a half-mask that occludes the lower part of the face, participants can extract facial information only from the unoccluded area. This forces them to change their fixation preference for facial identity recognition, resulting in longer reaction times. Previous eye-movement studies have shown that different participants have three different fixation preferences for the eyes, nose, and mouth when they recognize faces (Rennig et al., 2020), and that changes in fixation preferences affect facial identity recognition. In both the half-mask and no mask face identification conditions, participants could obtain information by looking at the eyes and mouth, whereas in the full-mask condition, since the participants could obtain information only from the eyes, the change in fixation preference led to an increase in the fixation counts and a longer reaction time.

In conclusion, mask occlusion influences identity recognition efficiency by altering cognitive processing strategies for facial recognition. Static facial recognition relies primarily on local processing strategies focused on the nose, whereas dynamic facial recognition tends toward holistic processing strategies. Mask occlusion, particularly full-face masks, compels adaptive shifts in processing strategies or intensifies local processing, as only the eyes remain visible under full-masks. Alternatively, feature weighting adjustments may occur; half-masks necessitate redistributing gaze. These strategic shifts increase cognitive load, manifesting as objective metric changes such as prolonged reaction times, elevated fixation counts and extended total fixation duration.

## 5. Conclusions

This study revealed that during the early stages of facial recognition, the self-advantage effect is confined solely to the visual modality. Furthermore, in the later stages of facial recognition, this effect is observed in both visual and audiovisual modalities; however, no such effect was observed in the auditory modality alone. In the later stages of dynamic facial recognition, mask occlusion causes greater interference in the recognition of others’ faces than in the recognition of one’s own faces. However, during the early stages and throughout the process of static facial recognition, mask occlusion does not significantly alter the degree of interference for either one’s own face or others’ faces. In both static and dynamic faces, voice recognition promotes masking effects during the early stages of facial recognition. However, in the later stages of facial recognition, this promotional effect of voice is only observed in dynamic faces. Moreover, the facilitating effect of voice on the distinction between oneself and others in dynamic facial recognition becomes apparent in the early stages of dynamic facial recognition and persists into the later stages. However, regardless of whether they were in the early or late stages of static facial recognition, the facilitation effect of voice did not differ between themselves and others. This study revealed that the cross-modal facilitative effect of auditory stimuli on visual processing is influenced by the self-advantage effect, which is observed only in dynamic face recognition.

## Figures and Tables

**Table 1:** Reaction time (ms, M ± SD) for different conditions in Experiment 1.

Mask Type	Self			Other		
	Face	Voice	Faces + Voices	Face	Voice	Faces + Voices
No mask	514 ± 42	588 ± 84	502 ± 51	548 ± 61	631 ± 81	541 ± 64
Full-mask	526 ± 52	591 ± 82	510 ± 55	564 ± 62	606 ± 83	540 ± 59
Half-mask	526 ± 58	595 ± 79	516 ± 52	571 ± 70	637 ± 82	559 ± 64

**Table 2:** Fixation counts (times, M ± SD), gaze duration (ms, M ± SD) and total fixation duration (ms, M ± SD) under different conditions in Experiment 1.

Mask Type	Type of Stimulus	Gaze Duration		Total Fixation Duration		Fixation Counts	
		Self	Other	Self	Self	Self	Other
No mask	Face	443 ± 53	469 ± 60	509 ± 44	539 ± 60	2.03 ± 0.47	2.06 ± 0.49
	F + V	436 ± 51	463 ± 63	515 ± 53	538 ± 66	1.88 ± 0.43	1.97 ± 0.53
Full-mask	Face	453 ± 56	488 ± 62	510 ± 41	551 ± 65	2.22 ± 0.50	2.44 ± 0.54
	F + V	438 ± 60	471 ± 63	506 ± 51	547 ± 64	2.04 ± 0.50	2.25 ± 0.50
Half-mask	Face	454 ± 70	493 ± 73	522 ± 63	572 ± 60	2.15 ± 0.51	2.30 ± 0.51
	F + V	443 ± 51	472 ± 66	513 ± 45	547 ± 63	1.97 ± 0.49	2.08 ± 0.46

Note: F + V refers to the condition of faces + voices.

**Table 3:** Reaction time (ms, M ± SD) for different conditions in Experiment 2.

Mask Type	Self			Other		
	Face	Voice	Faces + Voices	Face	Voice	Faces + Voices
No mask	517 ± 56	629 ± 78	516 ± 53	538 ± 54	609 ± 69	528 ± 54
Full-mask	535 ± 60	624 ± 72	525 ± 57	558 ± 68	608 ± 75	537 ± 62
Half-mask	530 ± 62	633 ± 87	521 ± 57	561 ± 63	617 ± 74	541 ± 63

**Table 4:** Fixation counts (times, M ± SD), gaze duration (ms, M ± SD) and total fixation duration (ms, M ± SD) under different conditions in Experiment 2.

Mask Type	Type of Stimulus	Gaze Duration		Total Fixation Duration		Fixation Counts	
		Self	Other	Self	Self	Self	Other
No mask	Face	455 ± 71	483 ± 51	542 ± 38	561 ± 46	1.84 ± 0.52	1.90 ± 0.50
	F + V	455 ± 63	454 ± 65	542 ± 42	555 ± 47	1.82 ± 0.48	1.82 ± 0.46
Full-mask	Face	479 ± 69	497 ± 66	554 ± 43	573 ± 49	2.17 ± 0.59	2.23 ± 0.57
	F + V	475 ± 66	480 ± 60	546 ± 42	557 ± 50	2.10 ± 0.55	2.09 ± 0.54
Half-mask	Face	469 ± 87	499 ± 62	554 ± 49	582 ± 51	1.96 ± 0.51	2.01 ± 0.52
	F + V	457 ± 73	469 ± 72	543 ± 46	566 ± 52	1.88 ± 0.49	1.94 ± 0.49

Note: F + V refers to the condition of faces + voices.

## Data Availability

The data that support the findings of this study are available from the corresponding author X.T. upon reasonable request. The design plan for the experiments was not preregistered on OSF. All data, scripts, and stimuli are available from the authors upon reasonable request.
